# Supplementary material for: Elevated HbA1c is associated with advanced brain age in severe obesity
Source: medRxiv. 2026 Jun 6:2026.06.04.26354935. Preprint. [Version 1] doi: 10.64898/2026.06.04.26354935 (PMC13252470; doi:10.64898/2026.06.04.26354935)
Supplement: Supplementary file 1 [file media-1.pdf]

SUPPLEMENTARY MATERIALS

Supplementary Figure 1. Cook's Distance and Leverage Plots for Linear Models

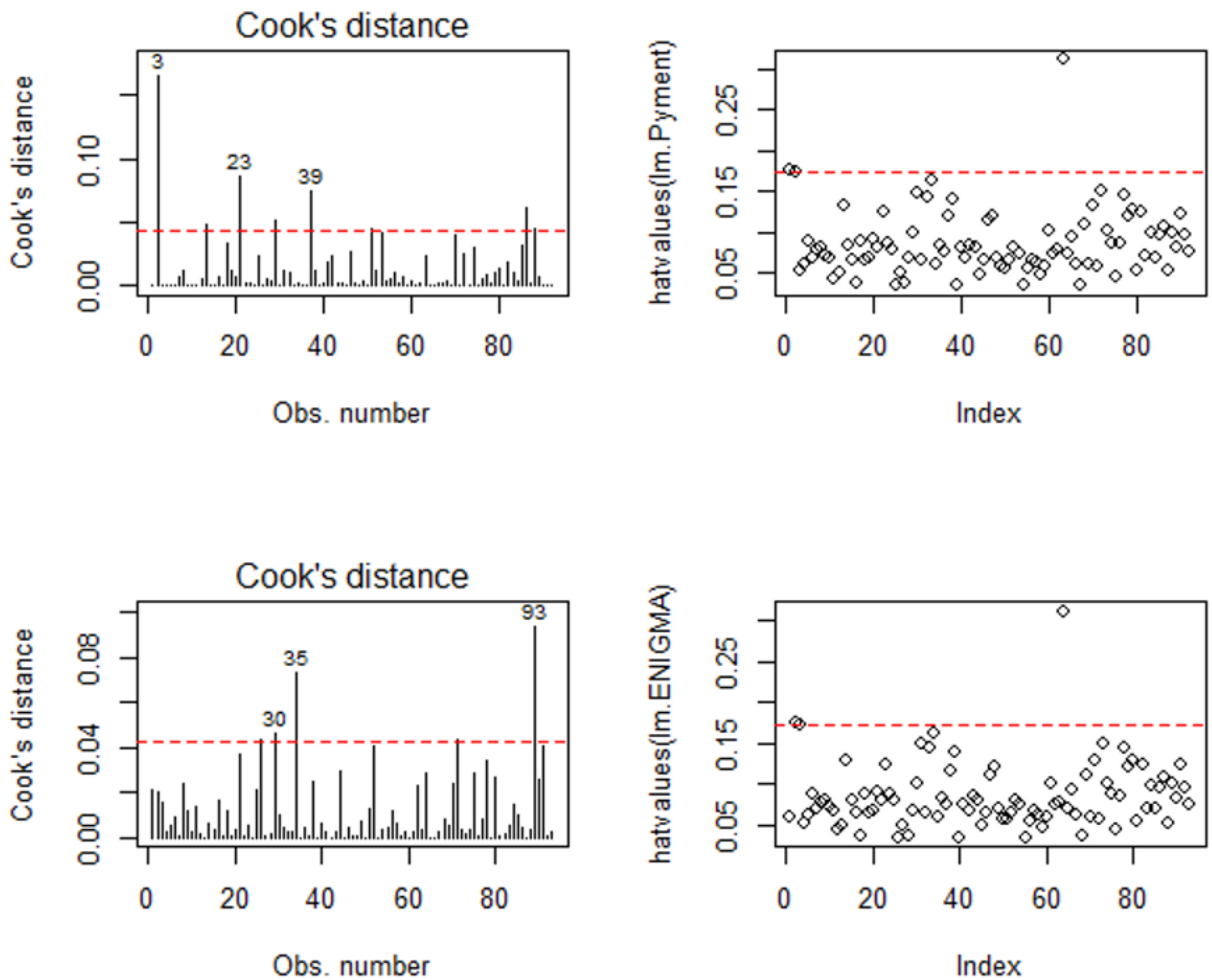

Supplementary Figure 1 shows the Cook's distance (left) and Leverage (right) plots for both the Pyment (top) and ENIGMA (bottom) linear models. Dotted line indicates cutoff values for outliers and high leverage points. Cook's distance plots indicated several outliers (values above 4/n) for both the ENIGMA and Pyment linear models. Leverage plots indicated several high leverage points (values above the 2p/n) for the ENIGMA and Pyment models.

Supplementary Figure 2. Added Variable Plots for BAG_{ENIGMA} Linear Model

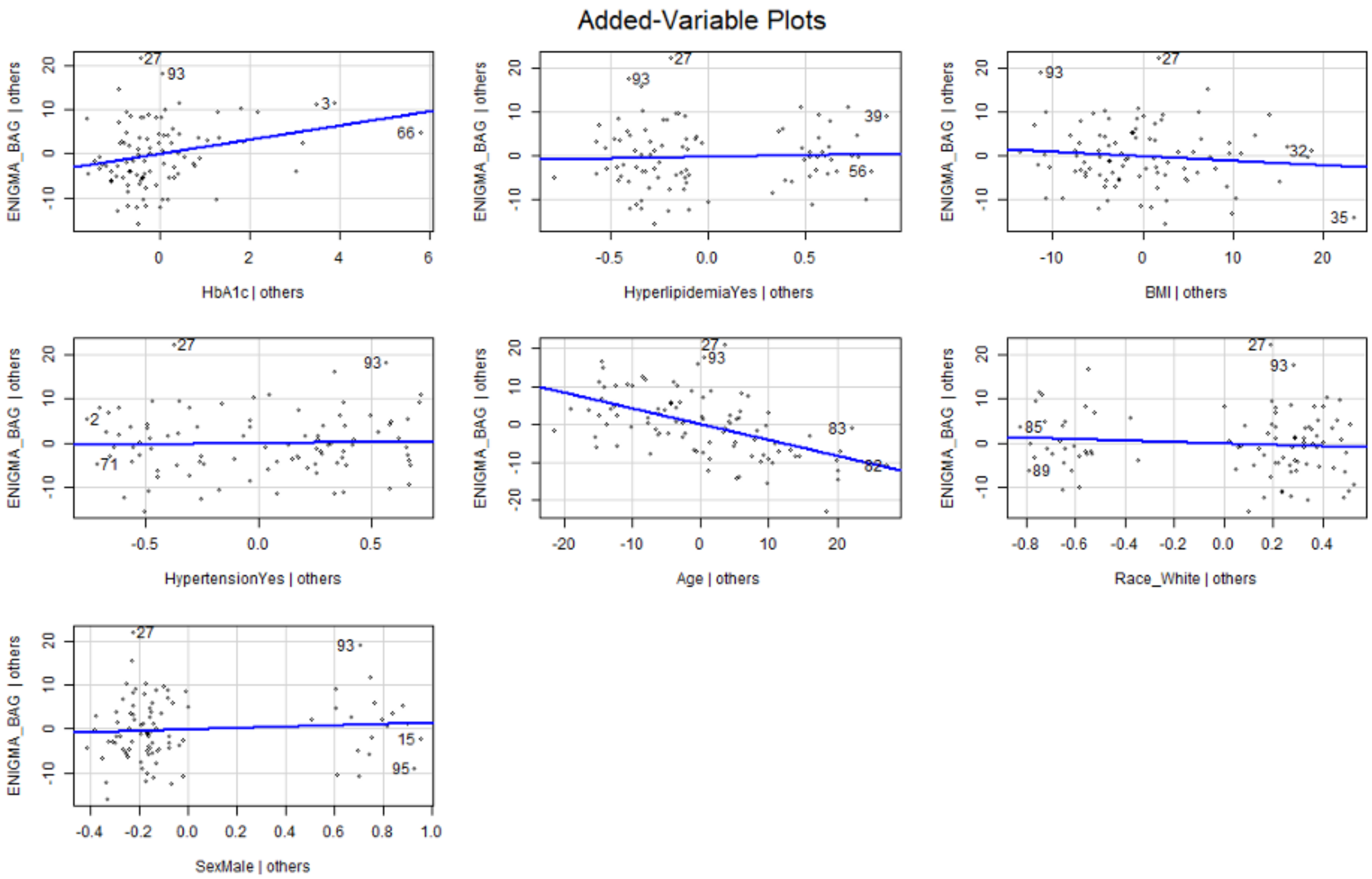

Added variable (partial regression) plots showing fitted partial regression lines between BAG and HbA1c, Hypertension, Hyperlipidemia, Age, BMI, Years of Education, Sex, and Race show the relationship between BAG_{ENIGMA} across individuals.

Supplementary Figure 3. Added Variable Plots for BAG_{Pyment} Linear Model

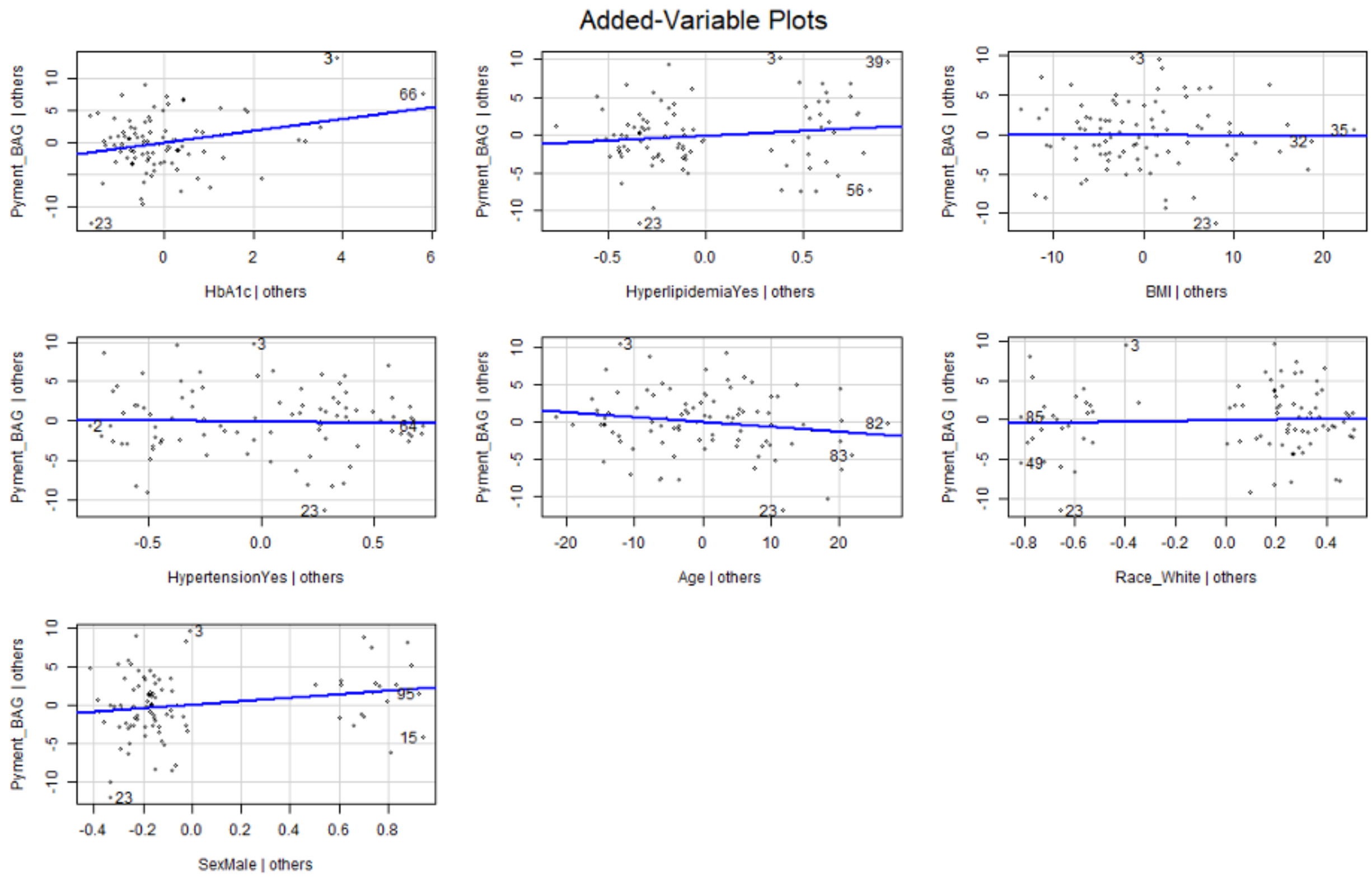

Added variable (partial regression) plots showing fitted partial regression lines between BAG and HbA1c, Hypertension, Hyperlipidemia, Age, BMI, Years of Education, Sex, and Race show the relationship between BAG_{Pyment} across individuals.

Supplementary Figure 4. Brain-Predicted Age Correlations with Chronological Age

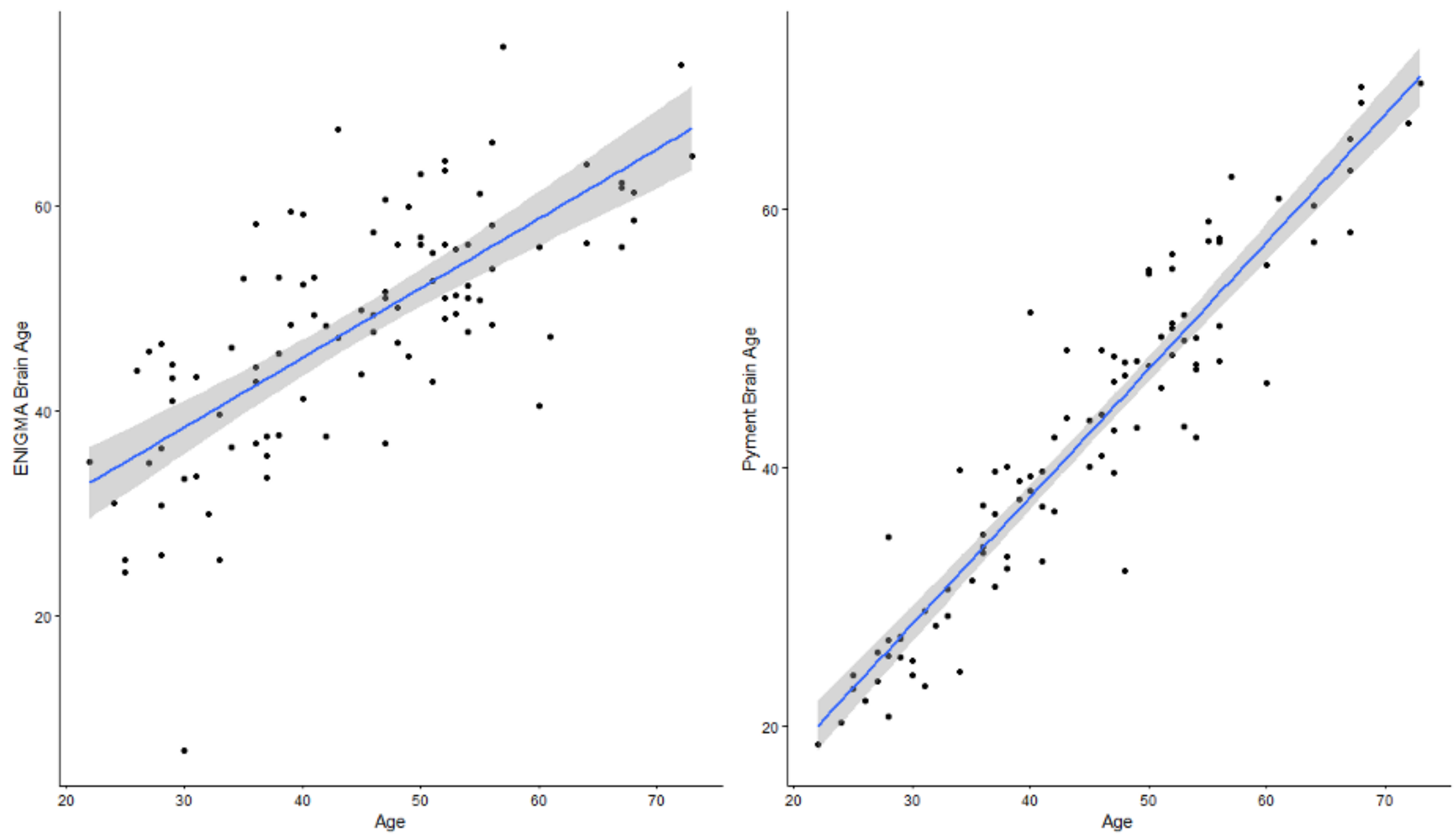

Scatterplots showing line of best fit for associations between brain predicted age and chronological age for ENIGMA ($\rho = .73$, $p < .001$) and Pyment ($\rho = .92$, $p < .001$) brain age value.

Supplementary Figure 5. Pyment Brain Age and ENIGMA Brain Age Scatterplot

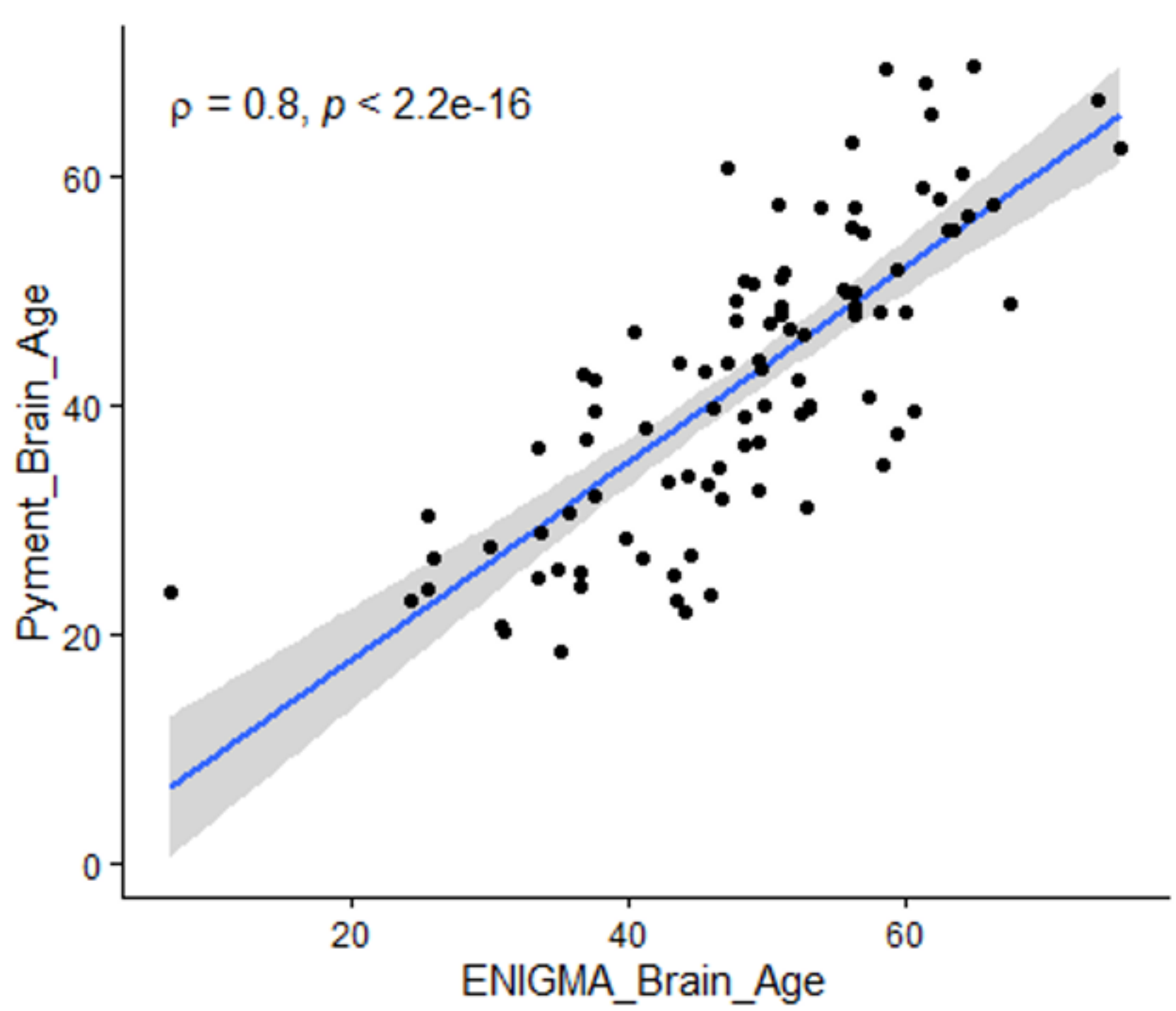

Scatterplot shows the relationship between brain-predicted age across individuals calculated by Pyment and ENIGMA models ($\rho = .80$, $p < .001$).

Supplementary Table 6. MM-Estimation Robust Linear Model for BAG_{Pyment} and BAG_{ENIGMA} with Categorical HbA1c

BAG_{Pyment}			
Factor	*B* Estimates	95% Confidence Intervals	*P* Values
Prediabetes	-1.04	[-2.82, 0.74]	.25
Diabetes	-1.62	[-3.93, 0.70]	.17
Uncontrolled Diabetes	3.47	[-0.09, 7.02]	.056
BMI	0.01	[-0.09, 0.10]	.89
Hyperlipidemia	1.11	[-1.06, 3.29]	.31
Hypertension	0.52	[-1.26, 2.29]	.56
Age	-0.02	[-0.09, -0.06]	.64
Sex (Male)	2.03	[-0.06, 4.12]	.057
Race (White)	0.30	[-1.48, 2.04]	.74
BAG_{ENIGMA}			
Factor	*B* Estimates	95% Confidence Intervals	*P* Values
Prediabetes	0.18	[-4.30, 4.67]	.93

Diabetes	0.09	[-3.92, 4.10]	.97
Uncontrolled Diabetes	7.99	[3.65, 12.33]	<.01*
BMI	-0.04	[-0.28, 0.18]	.67
Hyperlipidemia	1.08	[-1.85, 4.01]	.47
Hypertension	0.99	[-2.67, 4.66]	.59
Age	-0.39	[-0.52, -0.26]	< .01*
Sex (Male)	1.67	[-2.74, 6.08]	.33
Race (White)	-1.59	[-4.80, 1.62]	.45

*Indicates a statistically significant relationship. B denotes unstandardized linear regression coefficient estimates representing years. HbA1c was used to categorize diabetes groups into normoglycemic (< 5.7%,) prediabetes (5.7%-6.4%), diabetes (6.5%-8.0%) and uncontrolled diabetes (> 8.0%).
